# Visualization of Mitochondrial Ca^2+^ Signals in Skeletal Muscle of Zebrafish Embryos with Bioluminescent Indicators

**DOI:** 10.3390/ijms20215409

**Published:** 2019-10-30

**Authors:** Manuel Vicente, Jussep Salgado-Almario, Joaquim Soriano, Miguel Burgos, Beatriz Domingo, Juan Llopis

**Affiliations:** Physiology and Cell Dynamics Group, Centro Regional de Investigaciones Biomédicas (CRIB) and Facultad de Medicina de Albacete, Universidad de Castilla-La Mancha, C/Almansa 14, 02006 Albacete, Spain; Manuel.Vicente@uclm.es (M.V.); Jussep.Salgado@uclm.es (J.S.-A.); Joaquim.Soriano@uclm.es (J.S.); Miguel.Burgos@uclm.es (M.B.)

**Keywords:** aequorin, GFP-aequorin, mitochondria, calcium, zebrafish embryo, skeletal muscle, bioluminescence, genetically encoded calcium indicator (GECI), Twitch-4, microscopy

## Abstract

Mitochondria are believed to play an important role in shaping the intracellular Ca^2+^ transients during skeletal muscle contraction. There is discussion about whether mitochondrial matrix Ca^2+^ dynamics always mirror the cytoplasmic changes and whether this happens in vivo in whole organisms. In this study, we characterized cytosolic and mitochondrial Ca^2+^ signals during spontaneous skeletal muscle contractions in zebrafish embryos expressing bioluminescent GFP-aequorin (GA, cytoplasm) and mitoGFP-aequorin (mitoGA, trapped in the mitochondrial matrix). The Ca^2+^ transients measured with GA and mitoGA reflected contractions of the trunk observed by transmitted light. The mitochondrial uncoupler FCCP and the inhibitor of the mitochondrial calcium uniporter (MCU), DS16570511, abolished mitochondrial Ca^2+^ transients whereas they increased the frequency of cytosolic Ca^2+^ transients and muscle contractions, confirming the subcellular localization of mitoGA. Mitochondrial Ca^2+^ dynamics were also determined with mitoGA and were found to follow closely cytoplasmic changes, with a slower decay. Cytoplasmic Ca^2+^ kinetics and propagation along the trunk and tail were characterized with GA and with the genetically encoded fluorescent Ca^2+^ indicator, Twitch-4. Although fluorescence provided a better spatio-temporal resolution, GA was able to resolve the same kinetic parameters while allowing continuous measurements for hours.

## 1. Introduction

In skeletal muscle, calcium ions (Ca^2+^) released from the sarcoplasmic reticulum bind to troponin C and trigger acto-myosin crossbridge formation and force generation. They can also be taken up by the mitochondrial Ca^2+^ uniporter (MCU) [1,2,3], owing to the inner membrane potential difference [4]. Mitochondrial Ca^2+^, in turn, stimulates aerobic metabolism by activating the key enzymes of the Krebs cycle, the electron transport chain and oxidative phosphorylation [5]. ATP is used both for muscle contraction (myosin ATPase) and for relaxation (reuptake of Ca^2+^ into the sarcoplasmic reticulum by the SERCA ATPase). Increased ATP utilization lowers the ATP/ADP ratio, thus enhancing the rate of the respiratory chain (respiratory control). This functional interdependence between sarcoplasmic reticulum (SR) and mitochondria has a structural support, by the attachment of mitochondria to the Ca^2+^-release units in the SR [6,7]. This anchoring could lead to the generation of Ca^2+^ microdomains in space that facilitate Ca^2+^ uptake by the MCU. According to one study, mitochondrial Ca^2+^ oscillates in synchrony with cytoplasmic Ca^2+^ [8] to adjust mitochondrial energy production to the instantaneous metabolic needs of the muscle. Sarcoplasmic and mitochondrial Ca^2+^ have been studied mostly in myotubes and in isolated cells [9]. However, only a few reports have demonstrated this in situ in living organisms [8,10,11].

The purpose of this study was to visualize cytoplasmic and mitochondrial Ca^2+^ levels during spontaneous contractions of the trunk and tail of live zebrafish embryo with bioluminescent probes. The zebrafish (*Danio rerio*) is an exceptional vertebrate animal model with a high degree of genetic homology with humans, rapid ex utero embryonic development, and a relatively low cost. Skeletal muscle contractions in zebrafish start at 17 h post-fertilization (hpf) and intensify at 23 hpf [12]. Moreover, the embryos are transparent, which facilitates imaging, and genes can be expressed by cDNA/mRNA injection. To image zebrafish during development, non-invasive techniques, like bioluminescence, are preferred.

In our previous study, the Ca^2+^ sensitive photoprotein aequorin [13] was molecularly fused with yellow and red fluorescent proteins (FP), resulting in emission color changes by Förster resonance energy transfer (FRET) [14,15]. We recorded transient changes of Ca^2+^ associated with muscle contractions in zebrafish embryos during the segmentation period [14]. Ca^2+^ monitoring with aequorin (or FP-aequorin fusions) (see [16] for a review) is a well-established technique in which the photoprotein emits light when it binds to Ca^2+^ [13]. Since excitation light is not required, there is no phototoxicity, and recordings can be obtained for hours. However, the most important disadvantage of aequorin and its variants is their limited photon flow. Another drawback, which it shares with luciferases, is the need to provide exogenous substrate for reconstitution and the fact that the luminescence signal decays over time due to aequorin consumption. In contrast, genetically encoded Ca^2+^ indicators (GECIs) that work by fluorescence are brighter and easier to detect than luminescent probes, but excitation light causes autofluorescence, photobleaching and possibly phototoxicity, limiting the total imaging time. Both types of genetically encoded probes can be targeted to cellular organelles.

In this study, we established a model to study mitochondrial Ca^2+^ in vivo in zebrafish embryos. We imaged luminescence of GFP-aequorin (GA), expressed in the cytoplasm and trapped in the mitochondrial matrix (mitoGA) [17]. Transient expression of the probes was obtained by microinjection of mRNA in fertilized eggs at one cell stage, and aequorin was reconstituted by incubation of embryos with the substrate *f*-coelenterazine. Microscopy and functional tests confirmed that mitoGA was correctly targeted to mitochondria. We found that mitochondrial Ca^2+^ mirrors the sarcoplasmic changes in Ca^2+^ during spontaneous contractions through muscle development. In addition, Ca^2+^ transients propagated along the tail. We tracked the kinetics of cytoplasmic and mitochondrial Ca^2+^ by bioluminescence, and cytoplasmic Ca^2+^ with the genetically encoded fluorescent Ca^2+^ indicator, Twitch-4 [18].

## 2. Results

### 2.1. Characterization of the Expression of Cytoplasmic and Mitochondrial Biosensors

The luminescent Ca^2+^ indicators GA (not tagged) [19] and mitoGA (targeted to mitochondrial matrix) [17] were subcloned in the expression vector pCS2+, which allows transient high-level expression in vertebrate cells and in vitro transcription of sequences cloned in the poly-linker. The probes were transiently expressed by microinjection of mRNA in freshly fertilized zebrafish eggs. As these biosensors carry a fluorescent protein (GFP) in addition to the Ca^2+^-sensing aequorin, their expression and subcellular location was verified by fluorescence microscopy. Non-injected embryos were used as controls for fluorescence and development. GA expression was detected at 2 hpf in 99% of embryos, whereas mitoGA expression was observed only after 4 hpf. At 27–28 hpf, 95% of the injected embryos were fluorescent.

**Figure 1:**
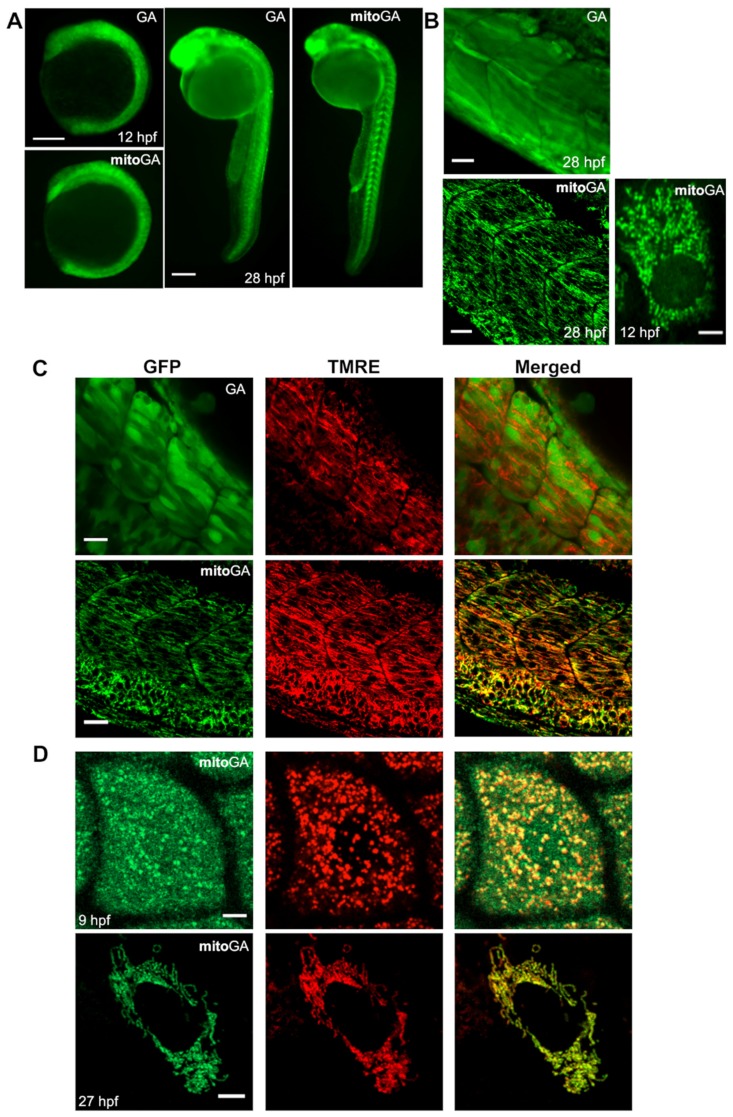
Expression of biosensors GFP-aequorin (GA) and mitoGA in zebrafish embryos. (**A**) Ubiquitous transient expression of GA and mitoGA in zebrafish at 12 and 28 hpf visualized with a fluorescence stereomicroscope. (**B**) Localization of GA and mitoGA expressed in the trunk musculature by confocal microscopy (28 hpf) and mitoGA in the enveloping layer (12 hpf). (**C**) MitoGA, but not GA, colocalizes with the mitochondrial marker TMRE in skeletal muscle at 28 hpf. (**D**) Colocalization of mitoGA with TMRE at 9 and 27 hpf in the cells of the enveloping layer of a zebrafish embryo. Mitochondrial import of mitoGA was complete at 27 hpf. The scale bars represent 200 µm (**A**), 20 µm (**B**,**C**) and 5 µm (**D**).

Figure 1A shows the fluorescence expression pattern of GA and mitoGA at 12 (3–6 somite stage) and 28 hpf on a dissecting microscope. The probes were expressed in many cell types in a mosaic pattern. At 28 hpf, in addition to autofluorescence of the yolk and labeling of the brain, fluorescence visible in the trunk and tail corresponded mostly to skeletal muscle.

The somites were well delimited by fluorescence. The overall fluorescence of mitoGA tended to be less than that of GA, as expected, because the mitochondrial matrix compartment has less volume than bulk cytoplasm.

For the goals of the study, it was crucial to ensure that mitoGA expression was restricted to mitochondria. We evaluated the expression of GA and mitoGA by confocal microscopy. Embryos expressing GA showed a fairly uniform labeling in skeletal muscle cells of the trunk, whereas the mitoGA embryos showed a filamentous staining, typical of mitochondria (Figure 1B). This suggests that the signal from human subunit VIII of cytochrome C oxidase used in mitoGA targets GA expression to the mitochondrial matrix in zebrafish.

To confirm the above conclusions, we performed colocalization experiments of GA and mitoGA with a mitochondrial chemical dye. The membrane potential dye TMRE is known to accumulate in respiring mitochondria, according to the Nernst equation, owing to its delocalized positive charge [4]. We first verified that TMRE labels mitochondria in zebrafish embryos. In 27 hpf embryos injected with TMRE at fertilization, cells displayed filamentous cytoplasmic red labeling and nuclear exclusion (Figure S1A). The uncoupler FCCP is known to cause redistribution of the TMRE cation to the cytoplasm and extracellular compartment. Thus, the filamentous TMRE labeling in trunk skeletal muscle cells largely disappeared within minutes of addition of FCCP (Figure S1B,C). This functional test suggests that in live zebrafish embryos, TMRE labels the mitochondrial matrix and that mitochondria were energized. Once labeling of TMRE in embryos was confirmed, we performed colocalization of mitoGA with TMRE by confocal microscopy. In embryos co-injected with TMRE and mitoGA mRNA, they co-localized in skeletal muscle cells of the trunk at 24–28 hpf (Figure 1C). Furthermore, mitochondria containing mitoGA were energized, since they accumulated TMRE. Accordingly, GA expression did not colocalize with TMRE.

Proteins endowed with mitochondrial targeting signals (like mitoGA), a sequence rich in basic residues, are synthesized in the cytoplasm and imported through the mitochondrial membranes into the matrix [20] and this process may limit their accumulation. We observed that in embryos co-injected with TMRE and mitoGA mRNA, cells of the enveloping layer at 9 hpf showed both mitochondrial and cytoplasmic labeling (Figure 1D, upper lane). This suggests that mitochondrial import of the biosensor was slower than its biosynthesis at that stage. However, at 27 hpf only mitochondrial labeling was observed (Figure 1D, lower lane): the balance between synthesis and mitochondrial import likely favored the latter at 27 hpf. This may be due to degradation of mRNA and/or maturation of the mitochondrial import machinery at this stage.

### 2.2. Monitoring Aequorin Reconstitution with Coelenterazine by Luminometry in Single Live Embryos

**Figure 2:**
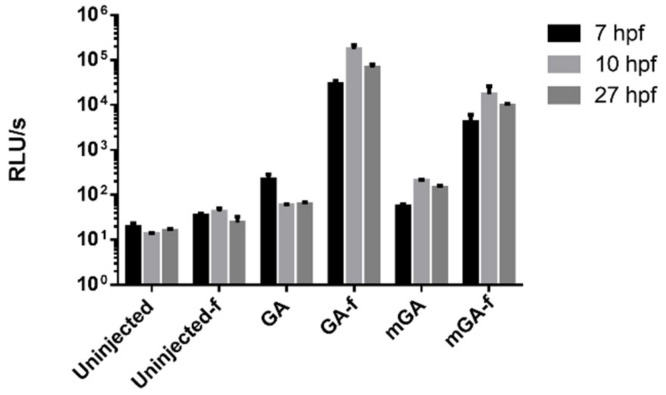
Aequorin reconstitution of GA and mitoGA (mGA) with *f*-coelenterazine assessed by luminometry in single embryos at 7, 10 and 27 hpf. Note the logarithmic scale. Shown as mean ± SEM (*n* = 6 embryos for each condition). The reconstituted GA-*f* and mGA-*f* were statistically different from all controls according to a one-way ANOVA test. RLU, relative luminescence units.

To determine the functionality of the aequorin biosensors as Ca^2+^ indicators in our experimental model, a test of maximum luminescence was performed on a microplate luminometer (Figure 2). Light release was measured in 7, 10 and 27 hpf embryos incubated with the substrate *f*-coelenterazine for 3, 6 and 23 h, respectively. Triton X-100 (0.5%) was added to break the plasma membrane, bringing aequorin into contact with extracellular Ca^2+^ and releasing all counts. GA reconstituted with *f*-coelenterazine showed a >1000-fold increase in emission compared to either un-injected embryos, un-injected embryos incubated with *f*-coelenterazine, or GA-injected embryos not incubated with the substrate. mitoGA embryos incubated with *f*-coelenterazine emitted about 100-fold more counts than their controls. This proves that aequorin can be reconstituted in live zebrafish embryos by incubation with *f*-coelenterazine. Moreover, functional aequorin was quite stable over time, since total counts were similar at 10 and 27 hpf. Mitochondrially trapped GA provided about 10-fold less luminescence than cytosolic GA.

### 2.3. Bioluminescence Imaging in the Trunk and Tail of Live Embryos Shows Two Periods of Spontaneous Ca^2+^ Activity in Cytosolic and Mitochondrial Compartments

Miller and coworkers have described two distinct Ca^2+^ signaling periods during the development of slow muscle cells within forming skeletal muscle in zebrafish embryos using transient ubiquitous expression of aequorin [21] or transgenic zebrafish that express apoaequorin in the musculature [22]. These periods of oscillating Ca^2+^ were found to occur between 17.5 and 19.5 hpf (signaling period 1 or SP1) and after 23 hpf (SP2), with a quiet period of about 3.5 h between them. The authors concluded that these temporal characteristics of Ca^2+^ signaling played a role in myocyte differentiation and myotome patterning in SP1. Both SP1 and SP2 periods correlated with muscle contractions, as was shown with Oregon green-1 dextran [22]. In this study, we examined whether mitochondrial Ca^2+^, measured with mitoGA, would mirror the sarcoplasmic changes in Ca^2+^ during early muscle differentiation.

**Figure 3:**
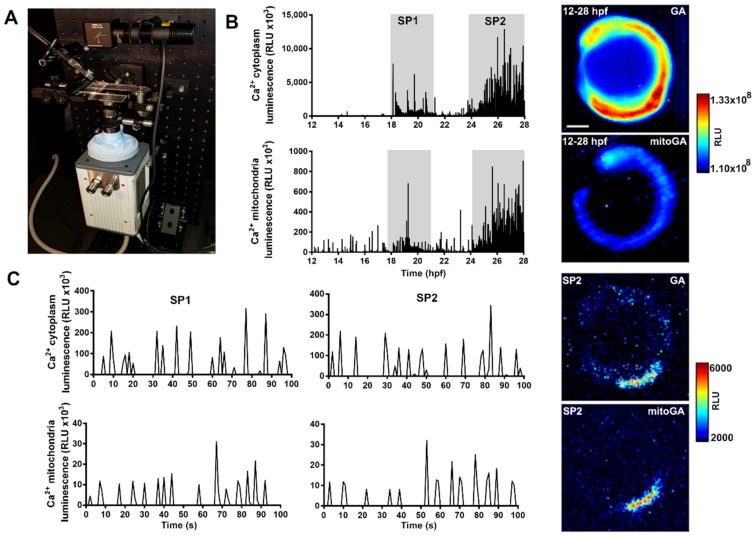
Continuous luminescence imaging of spontaneous Ca^2+^ transients in the trunk of zebrafish from 12 to 28 hpf. (**A**) Home-made low light microscope with EMCCD camera. (**B**) Representative experiments showing two periods of increased Ca^2+^ signaling, SP1 and SP2, in the cytoplasm and in mitochondria. Note the different luminescence scales. The images next to the graphs show the accumulated luminescence from 12 to 28 hpf. (**C**) Bioluminescence traces (100 s) corresponding to SP1 and SP2 in the cytoplasm (top graphs) and mitochondria (bottom graphs). The images on the right show one Ca^2+^ spike in the cytoplasm (top) and mitochondria (bottom) during the SP2 period. Images were acquired at 1 Hz. The scale bar represents 200 µm. The color scale indicates RLU.

For imaging experiments, a conventional fluorescence microscope adapted for luminescence was not sensitive enough to detect light emitted by GA or mitoGA, although images could be obtained at 1 Hz (1 s integration) in some embryos. To record luminescence images and increase the light gathering power, we constructed a low light microscope designed for bioluminescence and radioluminescence [23]. With this microscope (Figure 3A), brightness increased about 50-fold compared to the inverted microscope. Gathering more photons per pixel at the cost of spatial resolution, we could register bioluminescence images from all injected and reconstituted embryos at much higher acquisition rates, from 1 Hz up to 30 Hz (30 images/s).

Figure 3B,C show images and representative traces of GA/mitoGA luminescence in the trunk of embryos from 12 to 28 hpf. It is noteworthy that images were acquired continuously for 16 h, demonstrating that aequorin luminescence is a non-invasive technique. With the cytoplasmic probe GA, we recorded two periods of spontaneous Ca^2+^ signaling, corresponding to the SP1 and SP2 periods mentioned above. These two Ca^2+^ signaling periods were also observed in mitochondria with the biosensor mitoGA (Figure 3B,C), although usually, total counts were less than with embryos expressing GA. Thus, in these cells, sarcoplasmic Ca^2+^ transients or microdomains were of a sufficient magnitude for Ca^2+^ to be taken up by mitochondria. For the rest of the study, we focused on muscle contractions from 24 to 28 hpf.

### 2.4. Ca^2+^ Transients Measured with GA and mitoGA Reflect Contractions of the Trunk Observed by Transmitted Light

**Figure 4:**
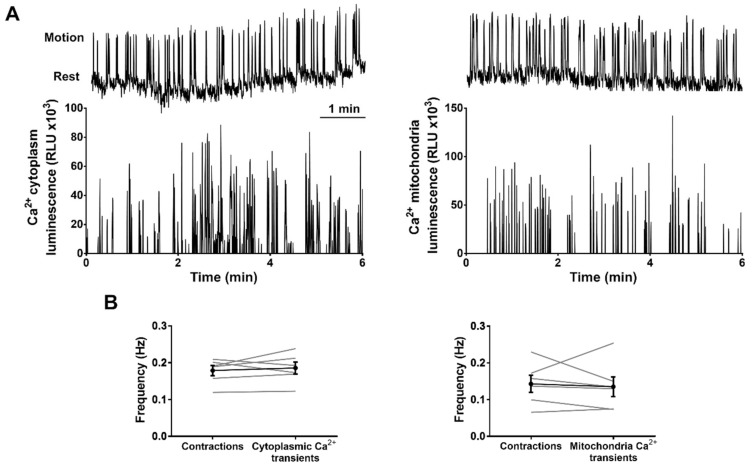
Frequency of spontaneous contractions and Ca^2+^ transients in the trunk of zebrafish embryos (24–28 hpf). (**A**) Representative experiments showing contractions (measured by transmission light, upper panels) and Ca^2+^ transients (measured by bioluminescence, lower panels) in the same embryo expressing GA (left) or mitoGA (right). (**B**) Comparison between the average frequency of contractions observed by transmitted light (imaging at 4.2 Hz) and Ca^2+^ transients (imaging at 4.7 Hz) in the cytoplasm (GA, left) and mitochondria (mitoGA, right). A two-tailed paired *t*-test was used. Shown as mean ± SEM (*n* = 6 embryos).

The issue of the low photon yield of aequorin luminescence relative to the sensitivity of the camera mentioned above posed the question of whether we were able to register Ca^2+^ transients for every contraction, or rather we only registered the largest Ca^2+^ transients. We examined the relationship between contractions in the trunk and tail, studied by transmitted light, and the Ca^2+^ activity recorded by luminescence imaging. It was not possible to register both events simultaneously with our setup, because any light in the light-tight box used for luminescence (see Section 4) would be picked up by the camera configured in its ultrasensitive mode. Thus, we imaged Ca^2+^ and contractions sequentially in the same embryos (expressing either GA or mitoGA) and compared their average frequencies. Figure 4A shows representative traces of contractions, identified by transmitted light (see Section 4), and Ca^2+^ signals obtained by bioluminescence (see also videos S1,S2). The average frequency of contractions and Ca^2+^ transients was not statistically different, both in the sarcoplasm (GA) and in mitochondria (mitoGA) (Figure 4B). Thus, in our conditions, these Ca^2+^ indicators were able to detect the Ca^2+^ transients in cytoplasm and in mitochondria corresponding to each contraction.

We examined whether Ca^2+^ transients in the SP2 period were caused by neural input. Tricaine is a reversible general anaesthetic commonly used in manipulation of zebrafish, which blocks neural action potentials (blocking voltage-dependent Na^+^ channels) but does not directly cause paralysis in skeletal muscle at standard concentrations [24]. Embryos treated with 1.1 mM tricaine did not contract or show any cytosolic Ca^2+^ transient, suggesting that these Ca^2+^ spikes were caused by neural input.

### 2.5. Functional Localization of mitoGA with Mitochondrial Inhibitors

The mitochondrial origin of the bioluminescence signals was demonstrated using two pharmacological inhibitors of mitochondria, the uncoupler FCCP and the recently described inhibitor of the MCU, DS16570511 [25], since mitochondrial depolarization or inhibition of the MCU should impair mitochondrial Ca^2+^ uptake.

**Figure 5:**
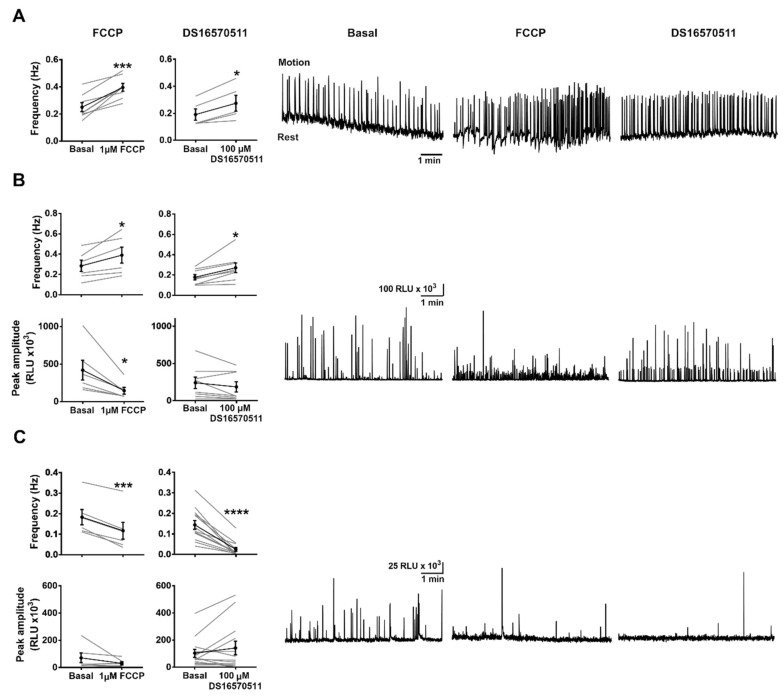
Effect of inhibiting mitochondrial Ca^2+^ uptake on the frequency of contractions and Ca^2+^ transients in 24–28 hpf embryos. Effect of the uncoupler FCCP (1 µM) and the MCU inhibitor DS16570511 (100 µM) on the frequency of skeletal muscle contractions in the trunk in non-injected embryos (**A**), and on cytosolic (**B**), and mitochondrial (**C**) Ca^2+^ transients. In (**B**) and (**C**) the frequency and amplitude of Ca^2+^ transients are shown. A two-tailed paired *t*-test was used. Shown as mean ± SEM (*n* = 5 to 13 embryos). The graphs on the right are representative experiments. Statistical significance was considered for * *p* < 0.05, *** *p* < 0.001, **** *p* < 0.0001.

We first checked the effect of 1 µM FCCP or 100 µM DS16570511 on contractions measured by transmitted light in control un-injected embryos during the SP2 period (24–28 hpf). Interestingly, after adding FCCP or DS16570511, the average frequency of contractions increased significantly by about 50% (Figure 5A). Since basal frequency was variable between embryos, the resting frequency was compared before and after the inhibitor in the same embryos (paired samples). Embryos treated with 1.1 mM tricaine to block neural input did not contract when treated with FCCP or DS16570511, indicating that the increase in frequency was dependent on the nervous system.

The effect of 1 µM FCCP and 100 µM DS16570511 on cytosolic Ca^2+^ transients was measured by GA luminescence. The frequency of sarcoplasmic Ca^2+^ transients increased by both compounds, in agreement with the increase in the frequency of contractions. However, the amplitude of Ca^2+^ transients decreased (FCCP) or did not change significantly (DS16570511) (Figure 5B). This increase in the frequency of contractions and cytosolic Ca^2+^ transients may be due to an effect on the motor pathway, and/or to a direct effect on Ca^2+^ release in myocytes (see Section 3).

In contrast with the effects on cytosolic Ca^2+^, both FCCP and DS16570511 decreased very significantly the frequency of mitochondrial Ca^2+^ transients (mitoGA luminescence) (Figure 5C). The amplitude of the Ca^2+^ transients did not change significantly with either compound, but it was difficult to determine with so few events.

The solvent DMSO, at the highest concentration used (0.2%) did not change the frequency of contractions in control un-injected embryos (Figure S2A,B), nor the frequency and amplitude of cytosolic (Figure S2C) and mitochondrial Ca^2+^ transients (Figure S2D). Furthermore, there was no statistically significant difference between the increase in frequency of contractions and Ca^2+^ transients for FCCP (Figure S3A) and DS16570511 (Figure S3B).

The above results support the conclusion that mitoGA is located in mitochondria, since FCCP, which collapses the membrane potential used by the uniporter to take up Ca^2+^ into the matrix [26], caused opposite effects on Ca^2+^ measured with GA and mitoGA. Furthermore, DS16570511, a compound recently described to inhibit the MCU [25], produced similar effects to FCCP.

### 2.6. Kinetic Parameters of Ca^2+^ Transients in the Cytoplasm

**Figure 6:**
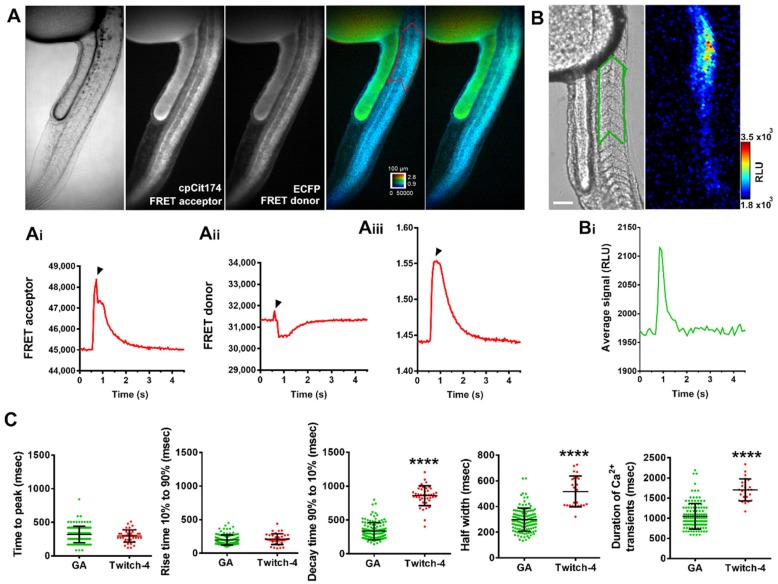
Kinetics of spontaneous Ca^2+^ transients in the trunk and tail in 28 hpf zebrafish embryos. (**A**) Representative experiment of an embryo expressing Twitch-4, imaged at 33 Hz. Left panels, transmitted light image and fluorescence FRET and donor images (cpCit174 and ECFP, respectively). Right panels, emission ratio of Twitch-4 fluorescence (FRET acceptor/donor) in pseudocolor, at rest and during the peak of a Ca^2+^ transient. The calibration square shows 100 µm, hue codes for emission ratio, and intensity codes for fluorescence. A region of interest (ROI, red trace) comprising somites 6–11 was used for quantification of FRET channel fluorescence (**Ai**), donor channel fluorescence (**Aii**) and fluorescence emission ratio (**Aiii**) during the Ca^2+^ transient. The black arrowheads indicate a motion artifact. (**B**) Transmitted light image and luminescence image during a Ca^2+^ spike in a GA expressing embryo. A ROI (green trace) was used for quantification of bioluminescence. (**Bi**) shows the Ca^2+^ transient measured in the ROI drawn in (**B**). The scale bar represents 100 µm. The color scale indicates RLU. (**C**) Comparison of the kinetic parameters of cytoplasmic Ca^2+^ transients measured with GA (bioluminescence imaged at 11.9 Hz, green points) and Twitch-4 (fluorescence acquired at 33 Hz, red points) in somites 6–11. A two-tailed unpaired *t*-test was used. Shown as mean ± S.D. (*n* = 128 transients from 3 embryos for GA; *n* = 25 transients from 4 embryos for Twitch-4). Statistical significance was considered for **** *p* < 0.0001.

Next, we characterized the shape and kinetics of the cytoplasmic Ca^2+^ transients with GA and with Twitch-4, a ratiometric fluorescent GECI [18]. To express Twitch-4, mRNA was injected into fertilized eggs at one cell stage, resulting in fluorescence, including the skeletal muscle of the trunk and tail in 24–28 hpf embryos (Figure 6A). 

Twitch-4 was imaged at 33 Hz with a wide-field fluorescence microscope. To image at this speed, the donor and acceptor FRET images (see Section 4) were acquired simultaneously with an image splitter. We routinely acquired 50 s of a high-speed video with continuous excitation of the donor. Longer exposures, however, resulted in appreciable photobleaching; 15 min illumination decreased 9% of the donor and 19% of the acceptor; thus, the ratio of FRET/donor images decreased. In addition, such long illumination could be phototoxic to the embryos. Figure 6A shows the individual FRET and donor fluorescence images and the Ca^2+^ rise (FRET/donor ratio images) during a spontaneous contraction of the trunk in a representative embryo. The Ca^2+^ rise was sharp and decayed in 1–2 s; the average kinetic parameters are shown in Figure 6C. Although the individual fluorescent traces show a motion artifact (black arrowheads) due to the contraction, the ratio corrects for that, a further example of the advantage of ratiometric dyes vs. intensiometric ones [27] in moving specimens.

The kinetics of the Ca^2+^ transients were also determined in GA-expressing embryos (Figure 6B). A bioluminescence image of one Ca^2+^ transient is shown. The kinetics of cytoplasmic Ca^2+^ spikes measured with GA (at 11.9 Hz) and Twitch-4 (at 33 Hz) were compared (Figure 6C). Rise parameters (time to peak and rise time 10–90%) were similar for both probes, but there were differences in the duration and decay parameters of the Ca^2+^ spikes. This was probably caused by the different signal dependence of the biosensors with Ca^2+^. The Hill slope of Twitch-4 is 1.04 [18], whereas aequorin luminescence changes in proportion to the 2.5 power of Ca^2+^ [28]. Thus, aequorin is not well suited to measure resting Ca^2+^ concentrations [29], since the signal is dominated by the higher concentration [28], whereas in Twitch-4, the fluorescence ratio changes in proportion to the 1.04 power of Ca^2+^ concentration. The decay of Twitch-4 fluorescence with Ca^2+^ (half time 0.5 s in vitro) [18] is not very different from that of aequorin luminescence (half time 0.4–0.8 s).

Twitch-4 was found to be a robust biosensor to characterize the kinetics of the Ca^2+^ transients with good time resolution (30 ms integration per image), whereas for GA, we used 84 ms integration per image (Figure 6). The spatial resolution with the sCMOS camera (binning 4 × 4), image splitter and objective used for fluorescence was 2.9 × 2.9 µm/pixel, whereas the EM-CCD camera (binning 4 × 4) and low light microscope provided a spatial resolution for luminescence of 12.8 × 12.8 µm/pixel. Nevertheless, GA was able to resolve the same kinetic parameters as Twitch-4 (Figure 6) while allowing continuous measurements for hours.

In bioluminescence imaging, the longer the integration, the better the signal-to-noise, at the cost of worsening the time resolution. For GA, acquisition frequencies at 4.7 Hz (213 ms integration time) allowed identification of all Ca^2+^ transients, since their frequency was the same as that of contractions (Figure 4). However, at 11.9 Hz (84 ms integration time), some events were not recorded (Figure S4); more events were lost at 30 Hz, likely being below the sensitivity threshold of the setup.

Figure S4 also shows the kinetic parameters of Ca^2+^ transients at various imaging sampling rates, to illustrate the limitations due to bioluminescence sampling. When imaging GA at a faster rate (30 Hz), only the largest Ca^2+^ peaks and the brightest portion of some transients were seen, and the kinetic parameters were artifactually shortened (Figure S4). In contrast, imaging at 4.7 Hz led to a slight overestimation of rise time 10–90%, since this parameter needed two points for determining the rate in Clampfit (see Section 4), and time to peak could not be well resolved (Figure S4). Thus, acquisition of 11.9 images/s was a good compromise to measure the kinetics of the Ca^2+^ transients by bioluminescence (Figure 6B,C).

### 2.7. Comparison of Kinetic Parameters of Ca^2+^ Transients in the Cytoplasm and Mitochondria

**Figure 7:**
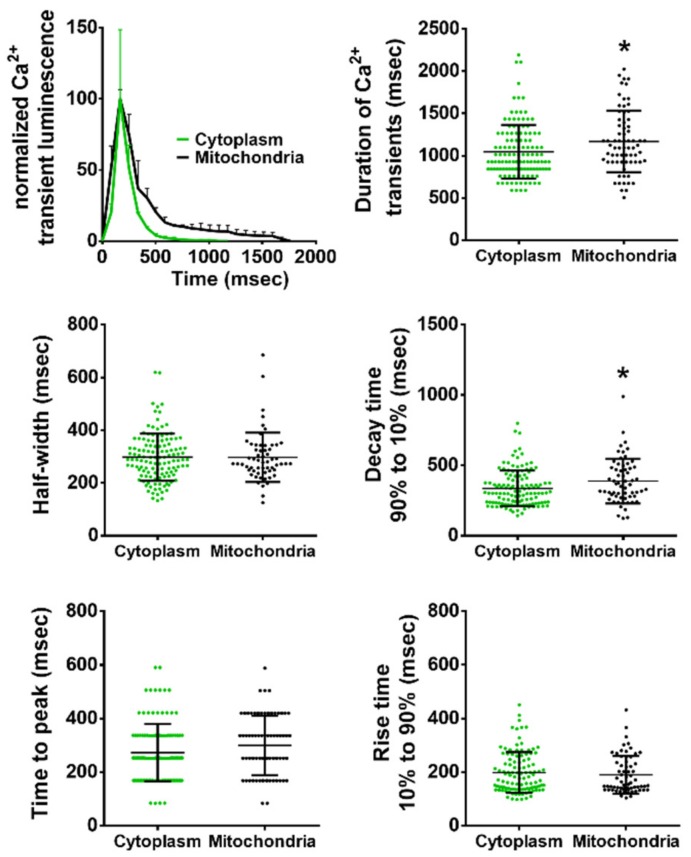
Comparison of the kinetic parameters of cytosolic and mitochondrial Ca^2+^ transients in zebrafish trunk in embryos expressing GA and mitoGA. Ca^2+^ transients in cytoplasm and mitochondria were compared. Embryos were imaged at 11.9 Hz. A two-tailed unpaired *t*-test was used. Shown as mean ± S.D. (*n* = 128 transients from 3 embryos for GA, green points; *n* = 71 transients from three embryos for mitoGA, black points). Statistical significance was considered for * *p* < 0.05.

We compared several kinetic parameters of the Ca^2+^ transients in the sarcoplasm and in mitochondria to elucidate possible differences between them. In embryos imaged at 11.9 Hz, there was no difference in the rise parameters (time to peak, rise time 10–90%), nor in half-width of the Ca^2+^ transients (Figure 7). However, Ca^2+^ spikes lasted longer in mitochondria than in the cytoplasm, as seen in the average traces. Accordingly, the decay time 90–10%, and the duration of the mitochondrial Ca^2+^ transients were significantly longer (Figure 7). Therefore, mitochondrial Ca^2+^ decay was delayed compared to the sarcoplasm.

Since GA and mitoGA could not be measured simultaneously in embryos, we could not determine whether there was a delay between the onset of cytosolic and mitochondrial Ca^2+^ transients [8].

### 2.8. Propagation of Ca^2+^ Along the Trunk and Tail

**Figure 8:**
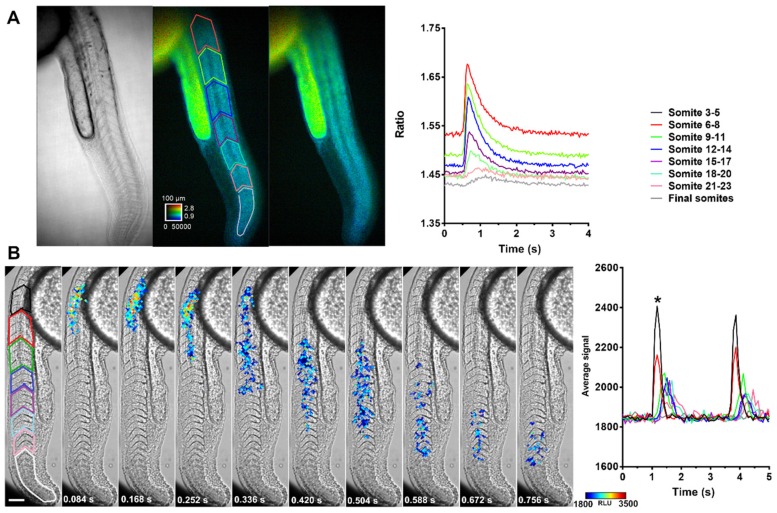
Propagation of Ca^2+^ transients along the trunk and tail in embryos expressing Twitch-4 and GA. (**A**) Transmitted light and emission ratio at rest and during a Ca^2+^ transient in a Twitch-4 expressing embryo at 28 hpf. The calibration square shows 100 µm, hue codes for emission ratio, and intensity codes for fluorescence. Fluorescence images were acquired at 33 Hz. ROIs were drawn over somites in groups of 3 (colored traces). The graph shows the time course of a Ca^2+^ transient in the indicated color-coded ROIs. (**B**) Snapshots of a Ca^2+^ transient in a GA-expressing embryo with luminescence overlaid on transmitted light images. ROIs were selected on the indicated somites in the left panel in groups of three (colored traces). Images were acquired at 11.9 Hz. The scale bar represents 100 µm. The color scale indicates RLU. The graph shows the time course of luminescence during two contractions on the indicated somites. The snapshots on the left correspond to the first contraction (*).

In the 24–28 hpf embryos, many spontaneous contractions observed by transmitted light seemed to propagate from trunk to tail. Figure 8A reveals the propagation of a Ca^2+^ transient measured with Twitch-4 in ROIs comprising three adjacent somites. The amplitude of the Ca^2+^ transient decreased rostro-caudally, and rise kinetics slowed down towards the tip of the tail (Figure 8A and video S3). Other contractions of smaller magnitude did not show such a clear propagation. Figure 8B shows the propagation of the Ca^2+^ signal along the trunk and tail in a GA-expressing embryo, with snapshots of luminescence at various times during the contraction (see also video S4). Although the time resolution of the fluorescent probe was about three-fold better than that of the luminescent one, both Twitch-4 and GA revealed propagating Ca^2+^ waves, which moved along the tail at about 1.6 mm/s.

## 3. Discussion

Aequorin has been used in the study of Ca^2+^ signaling in developing zebrafish and has contributed to elucidate many embryonic processes [22,30,31,32,33,34,35,36] (for a review see [37]). Due to its low photon yield, imaging studies with aequorin are always a challenge. In the present study, we tested the ability of GA to monitor cytosolic and mitochondrial Ca^2+^ signals during spontaneous skeletal muscle contractions in vivo. Compared to aequorin alone, the fusion GA permits the localization of the probe by fluorescence. In GA, GFP is fused to the N-terminus of aequorin, which results in a more stable and bright protein fusion [19]. In contrast, it was seen that GFP attached to the C-terminus of aequorin resulted in less luminescence than aequorin alone [21]. In fact, deletion or modification of its C-terminal proline residue has been shown to abolish luminescence [38,39]. Microinjection of GA mRNA in zebrafish embryos rendered ubiquitous and mosaic expression of the indicator. Although there was expression of the bioluminescent probes in other regions of the embryo (e.g., head), imaging enabled the selection of the trunk and tail, where the bulk of the signal came from skeletal muscle cells (Figure 1A). Cell-specific localization and homogeneous expression levels will be achieved with the generation of a transgenic line.

Whereas GA reported Ca^2+^ in the cytoplasm, for mitoGA to give a reliable measure of mitochondrial Ca^2+^, it was important to confirm maximal localization within the matrix (all mitochondria contain a functional amount of the probe) and minimal mistargeting (the probe was not expressed in any other compartment) [8,26,40]. In confocal images, the punctate/filamentous pattern and colocalization of mitoGA with TMRE ensured that most of the probe was confined in the organelle, and that new forming protein (cytosolic before translocation) was barely visible in the 24–28 hpf embryos (Figure 1B,C). It should be noted that mitochondrial import of the probe was not complete at earlier times of development (9 hpf, Figure 1D). In addition, functional studies with inhibitors of mitochondrial Ca^2+^ uptake supported that the biosensor was actually located within mitochondria; while contractions and cytosolic Ca^2+^ transients increased in frequency by these drugs, those in mitochondria decreased abruptly or disappeared (Figure 5). MitoGA has also been used in other organisms. Rogers et al. [11] generated a transgenic mice line with ubiquitous expression of mitGA (a similar fusion to mitoGA, but not identical). They showed mitochondrial Ca^2+^ increases by electrical stimulation of skeletal muscle, during spontaneous twitches in newborn mice, and under induction of epilepsy.

One particularity of aequorin is that apoaequorin needs to be reconstituted with its substrate coelenterazine to obtain functional photoprotein. In zebrafish embryos, coelenterazine can be simply added at high concentration (50 µM) to the bath [21]. However, Ca^2+^ rises occur spontaneously during aequorin reconstitution in zebrafish, leading to its consumption. Thus, if the burnout rate is faster than the reconstitution rate, aequorin will not be functional. Its luminescence is proportional to Ca^2+^ and to the amount of remaining functional photoprotein. We showed that after reconstitution of GA and mitoGA, embryos retained enough luminescence at 27 hpf to obtain a reliable signal for imaging experiments (Figure 2 and Figure 3).

As shown in Figure 3, bioluminescence was recorded continuously for 16 h, from 12 to 28 hpf, without signs of aequorin consumption. Experiments with an α-actin:aequorin transgenic zebrafish revealed two periods of Ca^2+^ activity in the trunk of 17–25 hpf developing zebrafish (SP1 and SP2), with a quiet period in between [22]. The cytoplasmic Ca^2+^ signals were associated to excitation-contraction coupling of the skeletal muscle cells in the trunk and tail, resulting in coordinated movements at different developmental stages [22]. Here, GA-recorded Ca^2+^ transients in long experiments (12 to 28 hpf) showed the same two periods of Ca^2+^ spikes with a similar frequency, although spikes were also observed in between SP1 and SP2 (Figure 3B). However, the frequency of cytosolic Ca^2+^ spikes and contractions at 24–28 hpf (0.02 Hz) increased to about 0.2 Hz when measured in short experiments (minutes) (Figure 3C and Figure 4). This higher frequency is in agreement with that of spontaneous movements reported in freely moving embryos (0.3 Hz at 24 hpf and a minimum of 0.08 Hz at 26 hpf) [12]. It is possible that the agarose restriction employed in long experiments and/or the embryo manipulation before imaging in short experiments account for this difference.

Interestingly, we showed that mitochondrial Ca^2+^ displayed the same distribution of Ca^2+^ signals, profiling SP1 and SP2. Moreover, the frequency of Ca^2+^ transients in cytoplasm and mitochondria was the same as that of contractions, measured by transmitted light (Figure 4). This suggests that for each contraction, cytosolic Ca^2+^ is taken up by mitochondria in myocytes. Other in vivo approaches in mice [8,11] and zebrafish [10] support this idea: a clear rise in mitochondrial Ca^2+^ was observed at each stimulation [8] or spontaneous contraction [10,11]. Our results add new evidence to the participation of mitochondria in shaping cytoplasmic Ca^2+^ during physiological contractions of skeletal muscle.

We found that the kinetics of mitochondrial Ca^2+^ rise were very similar to those of the cytosol, whereas its decay was slower (see the long tail of the mitochondrial signal in Figure 7). A similar conclusion was reported in hindlimb skeletal muscle in mice using mitGA [11]. In other reports, the decay of the mitochondrial Ca^2+^ concentration was much more delayed compared to the cytoplasmic levels. In mouse tibialis anterior muscle, it lasted tens of s [8], and in 50 hpf zebrafish skeletal muscle, a few minutes [10]. We did not see such a slow recovery of mitochondrial Ca^2+^ level to the baseline (Figure 3 and Figure 7). These authors employed the high affinity biosensors mitoYC2 [8], with an apparent dissociation constant (*K’d*) of 70 nM, and mitoYC2.60, with a *K’d* < 100 nM [10,41], although it has been revised to be 400 nM in vivo [42]. Moreover, YC2.60 is a slow indicator with a decay time constant of 5.24 s [42]. The affinity of mitoGA employed in this work and in [11] is close to 1 µM. It has been shown that mitochondria can accumulate Ca^2+^ to a high concentration upon physiological stimulation, about 500 µM in chromaffin cells [43] or 20–50 µM in Hela cells [44,45]. Thus, the importance of using biosensors of low Ca^2+^ affinity in order to record mitochondrial Ca^2+^ peaks has been pointed out [44]. Thus, the high affinity fluorescent probes [8,10] emphasize low, close to resting mitochondrial Ca^2+^ levels, whereas mitoGA ([11], this work) highlights higher concentrations.

The increase in the frequency of contractions and cytosolic Ca^2+^ transients with FCCP or DS16570511 (Figure 5) cannot be explained without further investigation. Drugs added to living embryos would act in many cell types, including neurons in the motor pathway or in the brain [12], disturbing their mitochondria and leading to the observed increase in contraction rate. Nonetheless, the direct effects of the drugs on the excitability or Ca^2+^ release in the myocytes expressing GA/mitoGA cannot be ruled out. Some authors have proposed that mitochondria, by taking up Ca^2+^ in domains close to the RyR clusters of the sarcoplasmic reticulum, and by regulating the redox environment, inhibit the onset of uncontrolled Ca^2+^ sparks and Ca^2+^-induced Ca^2+^ release (CICR) [6,7,46]. Blocking mitochondrial function with FCCP or DS16570511 would interfere with this mechanism.

To image developing or free behaving animals, minimally invasive techniques are a pre-requisite. Here, we demonstrated myoplasmic and mitochondrial Ca^2+^ transients with the bioluminescent sensors GA and mitoGA in prolonged experiments in developing zebrafish (Figure 3). A large area of the embryo, the trunk and tail, was imaged. Cytoplasmic Ca^2+^ signals possessed a steep rise and a slower descent. The time profile of single spontaneous contractions was well defined with GA at 11.9 Hz acquisition frequency (Figure 6, Figure 7 and Figure 8). In addition, the propagation of the contraction along the tail could be imaged, suggesting that all muscles in the tail participate in the movement. Most findings, including Ca^2+^ waves propagating along the tail, were consistent for GA and Twitch-4 biosensors.

In this study, we imaged physiological Ca^2+^ signals in the mitochondria of skeletal muscle with mitoGA in zebrafish embryos. Mitochondrial Ca^2+^ transients appeared for each contraction in 24–28 hpf embryos and their frequency and shape were similar to those of cytoplasmic Ca^2+^ spikes, with a slower mitochondrial decay. This is in agreement with previous reports in live mice [8,11] and zebrafish [10] showing that mitochondria take up Ca^2+^ with every contraction as a consequence of Ca^2+^ release from the sarcoplasmic reticulum. Tissue specific targeting of these probes will help to explore the relationship between cytoplasmic and mitochondrial Ca^2+^ in live animal models. In the future, the kinetic parameters shown here in control embryos will be compared with mutants defective in skeletal muscle function.

## 4. Materials and Methods

### 4.1. Constructs, Plasmids and mRNA Synthesis

The Ca^2+^ biosensors GFP-apoaequorin (GA) [19], Twitch-4 [18] (a kind gift of Dr. Oliver Griesbeck, Max Planck Institute of Neurobiology, Martinsried, Germany) and mitoGA [17] were cloned in the expression vector pCS2+ at the EcoRI/BamHI (GA, Twitch-4) or XhoI/XbaI (mitoGA) restriction sites. This vector has a Sp6 promoter for in vitro mRNA synthesis of genes inserted at the multiple cloning site. A single copy of the mitochondrial localization sequence of human cytochrome c oxidase subunit VIII [17,26] was used to direct import of the biosensor mitoGA into the mitochondrial matrix. All constructs were fully sequenced (Stab Vida, Caparica, Portugal).

The plasmids of the biosensors cloned in pCS2+ were linearized with the restriction enzymes NotI (GA, mitoGA) or HindIII (Twitch-4), and in vitro mRNA synthesis was performed with the mMESSAGE mMACHINE SP6 kit (Ambion, Austin, TX, USA) following the manufacturer’s protocol. mRNA resuspended in nuclease free water was quantified in a Nanodrop spectrophotometer and aliquots were made at 500 ng/µL and stored at −80 °C.

### 4.2. Collection and Maintenance of Zebrafish Embryos

Wild-type Tübingen zebrafish (*Danio rerio*) were kept in the Center for Animal Experimentation of the Albacete School of Medicine with a light/dark cycle of 14/10 h. Fertilized zebrafish eggs in synchronized stage were obtained following standard procedures and maintained in medium E3 (5 mM NaCl, 0.17 mM KCl, 0.33 mM MgSO_4_, 0.33 mM CaCl_2_, 0.002% methylene blue, pH 7.4 in double distilled H_2_O) at 28.5 °C. All animal procedures were carried out in compliance with UCLM Animal Experimentation Ethics Committee following national and EU regulations (approval identification code 900823 dated 30 May 2016, Consejería de Agricultura, Medio Ambiente y Desarrollo Rural, JCCM, Spain). All the experiments were conducted on zebrafish embryos and larvae of less than 120 hpf.

### 4.3. Microinjection of Eggs

Transient expression of the biosensors was obtained by injecting mRNA of GA, mitoGA or Twitch-4. Microinjection needles were prepared with glass capillaries of 1 mm external diameter (World Precision Instruments, Sarasota, FL, USA, catalog nº TW 100-F) in a horizontal puller (Model P-97, Sutter Instrument Co., Novato, CA 94949, USA) at 589 °C. Microinjection was performed manually on the blastodisc of one-cell stage eggs through the vegetal pole using a microinjection unit (Femtojet 4i, Eppendorf, Hamburg, Germany). The injection volume was 1–5 nL and the amount of mRNA was ~400 pg/egg. The injection solutions were diluted in 0.5% phenol red as an indicator of the injection volume. In some experiments, the mitochondrial chemical marker tetramethyl-ethylrhodamine (TMRE, 60 μM, Molecular Probes, Eugene, OR, USA) was injected together with mRNA of the Ca^2+^ biosensors, or by itself. The injected eggs were kept in an incubator at 28.5 °C for optimum development. The staging of the embryos was carried out as described [47].

### 4.4. Luminometry and Reconstitution of Aequorin In Vivo

A stock of *f*-coelenterazine (Biotium, Fremont, CA, USA) was prepared at 5 mM in methanol, 5 μL aliquots were prepared and frozen at −80 °C. To reconstitute functional aequorin, embryos injected with the mRNA of each biosensor were dechorionated at 4 hpf, transferred to a 24-well plate coated with agarose, and incubated (10 embryos per well) with 50 μM *f*-coelenterazine in E3 medium (28.5 °C in darkness) for 3–23 h, as indicated. To quantitatively determine the functional aequorin at different time points, total light emission from embryos of 7, 10 and 27 hpf was measured. Embryos were transferred to a 96-well opaque plate (1 embryo/well) in a microplate luminometer equipped with automatic injectors (Orion II, Titertek Berthold, Pforzheim, Germany). A Fast Kinetics protocol was designed with a total reading time of 1 min per well (1.2 s of light signal integration). An injection volume of 50 µL 0.5% Triton X-100 was added per well (0.25% final concentration) to trigger the release of luminescence.

### 4.5. Dechorionation and Mounting of Embryos for Microscopy

For the confocal and widefield fluorescence experiments, the embryos were dechorionated with fine forceps, embedded in 0.3% low melting point agarose in E3, preheated to 42 °C, and gelled on a 35-mm diameter glass bottom dish (ibidi, Gräfelfing, Germany). Once solidified, E3 medium was added. For the bioluminescence experiments, 8-well glass slides (ibidi) were used, and the embryos were embedded in 0.15% low melting point agarose in E3 medium. Embryos over 24 hpf were anaesthetized with 0.03% (1.1 mM) Tricaine (ethyl 3-aminobenzoate methanesulfonate salt, Sigma-Aldrich, Darmstadt, Germany) in E3 for 3 min prior to mounting for confocal microscopy. Embryos were not anesthetized for in vivo Ca^2+^ measurements.

### 4.6. Confocal Microscopy

Agar-embedded embryos were imaged in an inverse Axio Observer Zeiss LSM710 confocal system (Zeiss, Oberkochen, Germany) configured to use a Zeiss BiG-2 GaAsP detector and two detection channels. A green channel for GA or mitoGA, with 488 nm excitation laser and a 500–550 nm emission filter and red channel for TMRE, with 561 nm excitation laser and a 565–615 nm emission filter. Images were obtained with a PlanApo 10×/0.45 NA, oil immersion PlanN 40×/1.30 NA or oil immersion PlanApo 63×/1.40 NA objectives. For colocalization experiments, embryos coinjected with TMRE and GA or mitoGA were dechorionated and agar-mounted for observation as indicated above. Control embryos expressing only TMRE or GA were used to ensure the absence of bleed through (Figure S1A). Images were acquired with Zen2.3 software (Zeiss, Oberkochen, Germany).

### 4.7. TMRE Dissipation Experiments

Embryos injected with TMRE were dechorionated at 25 hpf and agar-mounted for observation under a confocal laser scanning microscope (LSM710, Zeiss, Oberkochen, Germany). A solution of 1 µM FCCP (Tocris, Bristol, UK) in medium E3 was added to the plate and fluorescence images were recorded at 0, 5 and 15 min.

### 4.8. Bioluminescence IMAGING with GA and mitoGA

The procedure for aequorin reconstitution described above was slightly modified. Fluorescent embryos were selected at 12 hpf, dechorionated and incubated in the dark at 28.5 °C with 50 µM *f*-coelenterazine in 0.5 mL of E3 medium (10 embryos per well). At 12 hpf (for overnight experiments) or at 24 hpf, embryos were embedded in 0.15% low melting point agarose and transferred to a 8-well glass slide (ibidi). Bioluminescence images were acquired with a custom-built microscope (Low Light Microscope, [23]) with an EM-CCD (512 × 512 pixels, EMC9100-13, Hamamatsu Photonics, Hamamatsu, Japan) as detector, placed at the microscope bottom. All components required to build the microscope were from Thorlabs GmbH (Newton, NJ, USA). The microscope was equipped with a 4× CFI PlanApo Lambda air objective (Nikon, Tokyo, Japan) used as the tube lens, and a 20× Nikon CFI PlanApo Lambda air objective (0.75 NA). The magnification of these combined lenses (*f* tube lense/*f* objective lens) was 5×. This microscope was equipped with a LED lamp for transmitted light. The microscope was housed in a light-tight box to maintain complete darkness during bioluminescence imaging. The embryos were maintained at 28 °C. The bioluminescence images were acquired continuously in 16 bits with 4 × 4 binning, 255 camera gain, at a rate of 1, 4.7, 11.9 or 30 Hz (frames/s). With this configuration, the resolution of the images was 12.8 µm × 12.8 µm/pixel and the total field of view (FOV) was 1638.4 µm × 1638.4 µm. Transmitted light images were acquired at 4.2 Hz to correlate the luminescence with the anatomical structures of the embryo, but they were not acquired simultaneously with luminescence.

To assess biosensor expression levels prior to bioluminescence experiments, fluorescence images of the embryos were acquired in a stereomicroscope (MZ10F Leica, Wetzlar, Germany) with a CCD camera (ORCA-ER, Hamamatsu Photonics) controlled with AquaCosmos2.6 (Hamamatsu Photonics). The microscope was equipped with a HC-YFP cube (HC500/24 excitation filter, 520 nm beamsplitter and HC542/27 emission filter).

### 4.9. Treatment with Mitochondrial Inhibiting Drugs

Embryos basal contractions were registered for 15 min, observation was briefly interrupted to add 1 µM Carbonylcyanide-p-trifluoromethoxyphenylhydrazone (FCCP, Tocris), or 100 µM DS16570511 (Caiman Chemical, Ann Arbor, MI, USA) and resumed for 15 min.

### 4.10. Detection of Contractions with Transmitted Light

All the contractions shown were spontaneous, occurring naturally during development of skeletal muscle. No external stimulation was employed. Time-lapses of embryo trunk contractions were recorded in the Low Light Microscope configured for transmitted light (see Bioluminescent imaging above). Videos in TIFF format were analyzed and processed in Fiji-ImageJ (NIH) [48]. For detection of contractions, two identical ROIs were placed close to each other on a somite in the embryo trunk and outside the embryo. The ratio of their intensity changed whenever a contraction occurred.

### 4.11. Bioluminescence Image Analysis and Processing

Ca^2+^ transients were differentiated from background by means of their location, intensity and size. Signals located over the embryo trunk, being larger than 9 adjacent pixels and brighter than a minimum intensity value, were considered a Ca^2+^ transient. The minimum intensity value was set to each frame taking into account a background analysis. To perform this analysis, two types of backgrounds were considered: general background (GB) and embryo background (EB). GB considers changes in detector noise over time; it was calculated on each individual frame by analyzing an artifact-free area in the medium surrounding the embryo. EB takes into account coelenterazine spontaneous bioluminescent emission; it was calculated once in each experiment by analyzing 15 different frames at times when no visible Ca^2+^ transients took place.

Image analysis and processing was automated through a FIJI macro (available upon request). The macro interacts with a user through a variety of graphical user interfaces that require: 1) drawing the region of the embryo to be analysed (ROI1), 2) moving the drawn region to an artifact-free area out of the embryo (ROI2) and 3) choosing 15 frames with no visible Ca^2+^ transients. GB was calculated as GB = Mean pixel value in ROI2 + 2*S.D. (S.D. standard deviation). EB was calculated as:

(1) $$\mathrm{EB}=\frac{\sum_{i=15}^{i=1}\left[Mean\;pixel\;value\left(\mathrm{ROI}1\right)-Mean\;pixel\;value\left(\mathrm{ROI}2\right)\right]}{15}+2\times S.D.$$

A final threshold was set to each frame adding GB and EB. The integrated density of Ca^2+^ transients was calculated for each frame and imported into Clampfit for event analysis. For characterization of Ca^2+^ spike kinetics, data were analyzed with Clampfit 10.7 (Molecular DevicesSan José, CA, USA), and the following parameters were determined: frequency, time to peak, peak amplitude, rise time 10% to 90%, decay time 90% to 10%, half-width (the width in s at half height), and duration of the event.

### 4.12. Ratiometric Fluorescence Imaging with Twitch-4

We acquired fluorescent images of the trunk and tail in 24–28 hpf embryos expressing the ratiometric fluorescent biosensor Twitch-4 [18] with a wide-field fluorescence microscope (DMIRE-2, Leica,) equipped with a sCMOS camera (2048 × 2048 pixels, ORCA-Flash 4.0, Hamamatsu Photonics), controlled by the software Aquacosmos 2.6 (Hamamatsu Photonics). The image acquisition rate was 33 Hz (30 ms image integration) in experiments lasting 50 s. To image at this speed, the donor (ECFP) and acceptor (cpCitrine174) FRET images were acquired truly simultaneously with an image splitter (W-View Gemini, Hamamatsu Photonics), which allows dividing the camera field in two halves. This was important for correcting motion artifacts during embryo contractions. The embryos were heated with a chamber incubator (Pecon, Erbach, Germany).

The embryos were excited continuously with a LED source (Lambda TLED+, Sutter Instrument) using a 440AF21 nm bandpass filter (Chroma, Bellows Falls, VT, USA) and a beamsplitter (455DLRP, Omega Optical, Brattleboro, VT, USA). Fluorescence passed through an image splitter with emission filters 483/32 nm and 542/27 nm (Semrock, Rochester, NY, USA), separated with beamsplitter 509-FDi01 (Semrock). A 10× air objective (HC PlanApo 0.45 NA, Leica) and binning 4 × 4 were used. With this configuration, the resolution of the images was 2.9 µm × 2.9 µm/pixel and the total field of view (FOV) after image splitting was 742.4 µm (H) × 1484.8 µm (V). Transmission images were acquired to correlate fluorescence with the anatomical structures of the embryo.

The FRET image corresponds to cpCitrine174 emission (542 nm channel) with 440 nm excitation, and the donor image corresponds to ECFP emission (483 nm channel) with 440 nm excitation. The ratio FRET/donor image was computed pixel by pixel. For ratio image processing, we used the custom-made software Ratioscope, programmed by Dr. Pierre Vincent (CNRS, Sorbonne University, Paris, France) on IgorPro 8 (WaveMetrics, Lake Oswego, OR, USA), and program Clampfit 10.7 (Molecular Devices) for extracting the kinetic parameters of Ca^2+^ transients.

### 4.13. Statistical Analysis

A one-way ANOVA test was used to compare groups of embryos where indicated with a Bonferroni *post-hoc* test for paired comparisons. A two-tailed *t*-Student test was used for paired or independent samples, as indicated. Data are shown as the mean ± standard error of the mean (SEM) or standard deviation (S.D.), as indicated. Statistical significance was considered for * *p* < 0.05, ** *p* < 0.01, *** *p* < 0.001, **** *p* < 0.0001. Data were analyzed in Graphpad Prism 6 (Graphpad Software, San Diego, CA, USA).

## Supplementary Materials

Supplementary materials can be found at https://www.mdpi.com/1422-0067/20/21/5409/s1.

## Abbreviations

cpCitrine174	Citrine fluorescent protein circularly permuted at amino acid 174
DMSO	Dimethyl sulfoxide
ECFP	Enhanced cyan fluorescent protein
FCCP	Carbonylcyanide-*p*-trifluoromethoxyphenylhydrazone
FOV	Field of view
FP	Fluorescent protein
FRET	Förster resonance energy transfer
GA	GFP-aequorin
GECI	Genetically-encoded calcium indicator
GFP	Green fluorescent protein
hpf	Hours post-fertilization
MCU	Mitochondrial Ca^2+^ uniporter
mitoGA	Mitogfp-aequorin trapped in the mitochondrial matrix
RLU	Relative luminescence unit
ROI	Region of interest
SP1	Signaling period 1
SP2	Signaling period 2
TMRE	Tetramethylethylrhodamine
