# Supplementary material for: Visualization of Mitochondrial Ca2+ Signals in Skeletal Muscle of Zebrafish Embryos with Bioluminescent Indicators
Source: Int J Mol Sci. 2019 Oct 30;20(21):5409. doi: 10.3390/ijms20215409 (PMC6862566; doi:10.3390/ijms20215409)
Supplement: Supplementary file 1 [file ijms-20-05409-s001.zip › Vicente et al 2019 Supplementary Material.docx]

Article

Visualization of mitochondrial Ca^2+^ signals in skeletal muscle of zebrafish embryos with bioluminescent indicators

Manuel Vicente^1^, Jussep Salgado-Almario^1^, Joaquim Soriano^1^, Miguel Burgos^1^, Beatriz Domingo^1,^ * and Juan Llopis^1,^*

^1^ Physiology and Cell Dynamics. Centro Regional de Investigaciones Biomédicas (CRIB) and Facultad de Medicina de Albacete, Universidad de Castilla-La Mancha, C/ Almansa 14, 02006 Albacete, Spain; [Manuel.Vicente@uclm.es](mailto:Manuel.Vicente@uclm.es)

***** Correspondence: [juan.llopis@uclm.es](mailto:juan.llopis@uclm.es); [beatriz.domingo@uclm.es](mailto:beatriz.domingo@uclm.es); Tel.: +34-967 599 315 (J.L.)

Received: date; Accepted: date; Published: date

1. SUPPLEMENTARY FIGURES

| 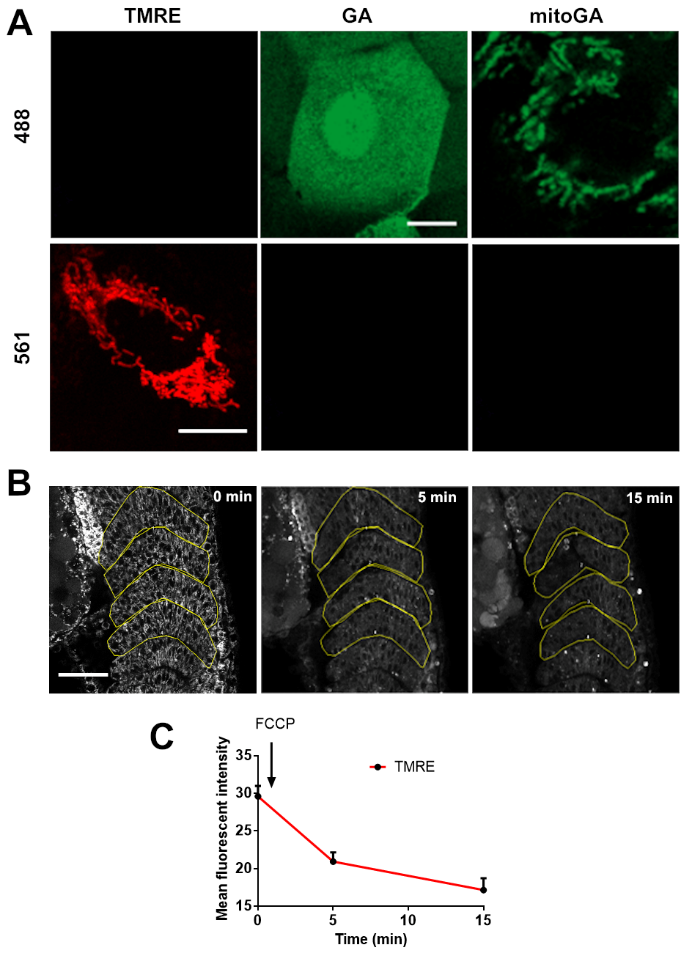 |
| --- |

**Figure S1.** Mitochondrial localization of TMRE and mitoGA in zebrafish. A) Emission of TMRE, GA and mitoGA into green and red channels (with 488 nm and 561 nm excitation, respectively) in a confocal microscope (27 hpf embryo). B) Dissipation of mitochondrial TMRE labeling by the uncoupler FCCP in a 28 hpf embryo. Images of zebrafish trunk were acquired before, 5 and 15 min after FCCP addition. C) The average fluorescence of the somites in the ROIs indicated in B) was quantified over time. Scale bar 10 µm (A) and 50 µm (B).

| 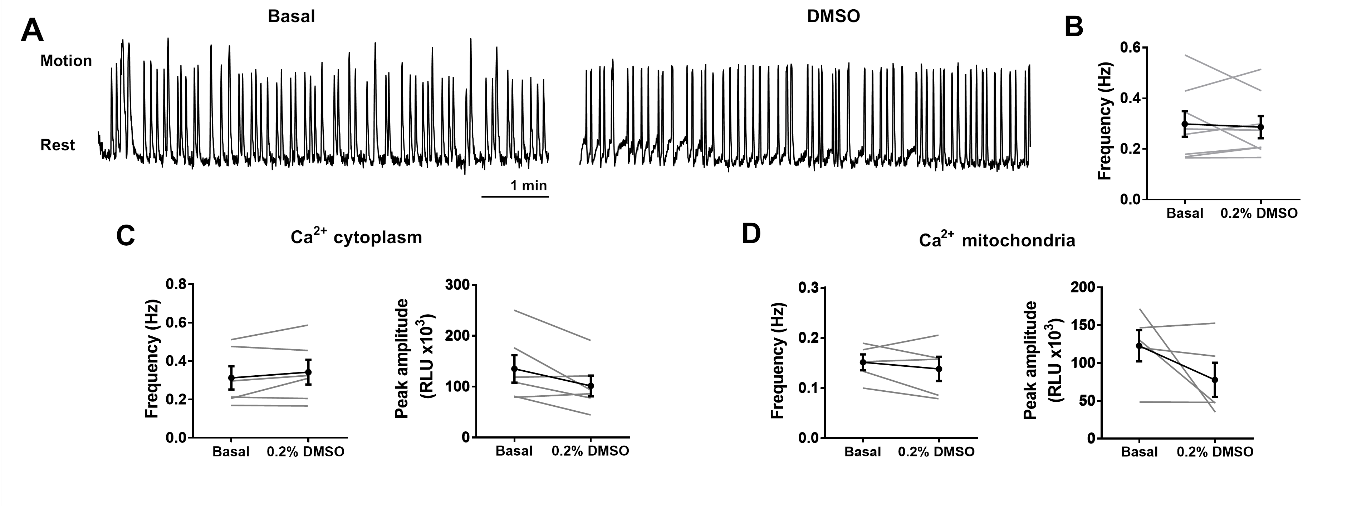 |
| --- |

**Figure S2.** Effect of the solvent DMSO (0.2%) on the frequency of contractions measured by transmitted light (A, representative experiment; B), and on the frequency and amplitude of the Ca^2+^ transients in the cytoplasm (C) and mitochondria (D). Two-tailed paired *t*-test was used. Shown as mean ± SEM (n=5 to 8 embryos).

| 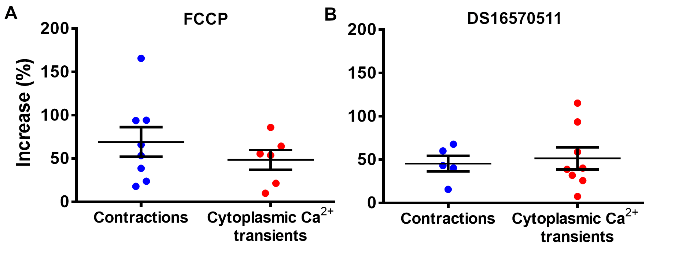 |
| --- |

**Figure S3.** Comparison between the increase in frequency of contractions and that of cytoplasmic Ca^2+^ transients, triggered by 1 µM FCCP (left) or by 100 µM DS16570511 (right). Two-tailed unpaired *t*-test was used. Shown as mean ± SEM (n=5 to 8 embryos).

| 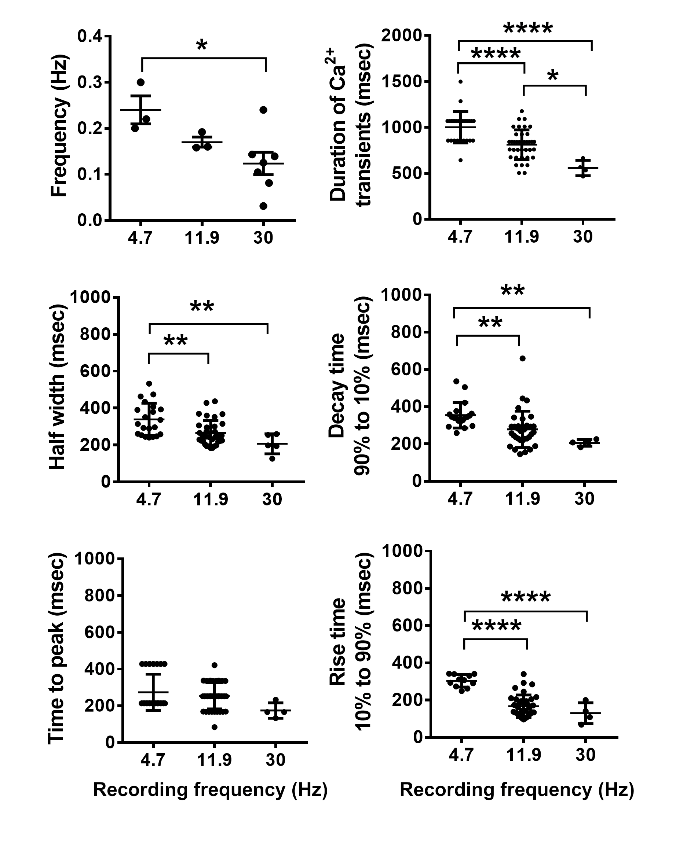 |
| --- |

**Figure S4.** Kinetic parameters of Ca^2+^ transients measured at different imaging sampling rates (4.7, 11.9 and 30 Hz) in the same embryo expressing GA in the cytoplasm. One-way ANOVA statistical test was used. Shown as mean ± S.D. (n=3 to 6 embryos for the frequency of Ca^2+^ transients; for the rest of the graphs n=4 to 38 Ca^2+^ transients in the same embryo).

2. SUPPLEMENTARY VIDEOS

**Video S1.** Cytosolic Ca^2+^ transients in a 27 hpf embryo expressing GA. The bioluminescence intensity is shown in pseudocolor scale, overlaid to a brightfield image.

**Video S2.** Mitochondrial Ca^2+^ transients in a 27 hpf embryo expressing mitoGA. The bioluminescence intensity is shown in pseudocolor scale and overlaid to a brightfield image.

**Video S3.** Cytosolic Ca^2+^ transient and propagation during a single contraction in a 28 hpf embryo expressing Twitch-4. The emission ratio is shown in pseudocolor scale.

**Video S4.** Rostro-caudal propagation of cytosolic Ca^2+^ transients in a 27 hpf embryo expressing GA. The bioluminescence intensity is shown in pseudocolor scale and overlaid to a brightfield image.

| 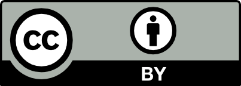 | © 2019 by the authors. Submitted for possible open access publication under the terms and conditions of the Creative Commons Attribution (CC BY) license (http://creativecommons.org/licenses/by/4.0/). |
| --- | --- |
