# Supplementary figures and images for: Visualization of Mitochondrial Ca2+ Signals in Skeletal Muscle of Zebrafish Embryos with Bioluminescent Indicators
Source: Int J Mol Sci. 2019 Oct 30;20(21):5409. doi: 10.3390/ijms20215409 (PMC6862566; doi:10.3390/ijms20215409)

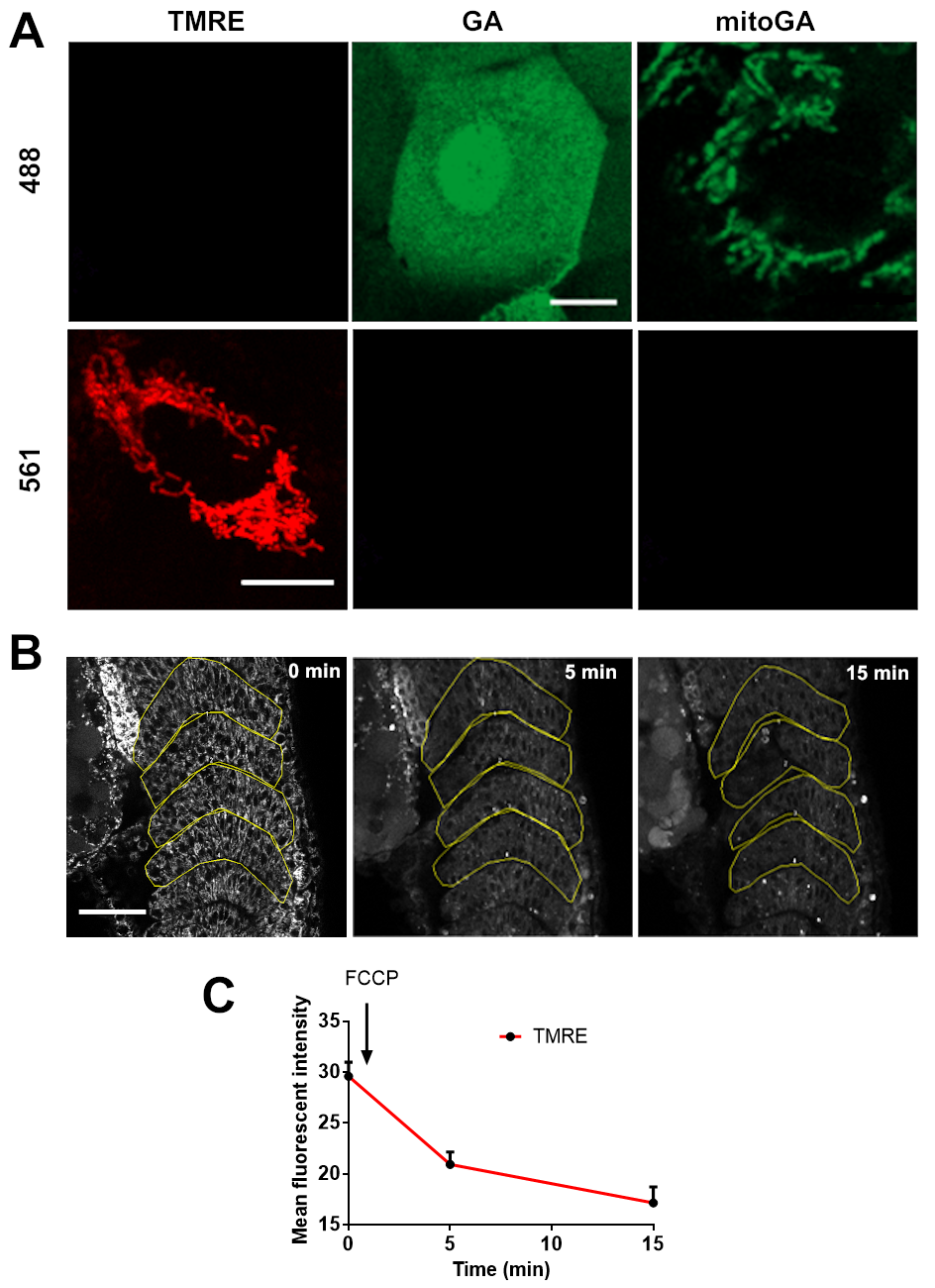

Supplement: Supplementary file 1 [file ijms-20-05409-s001.zip › Figure Supp1.tif]

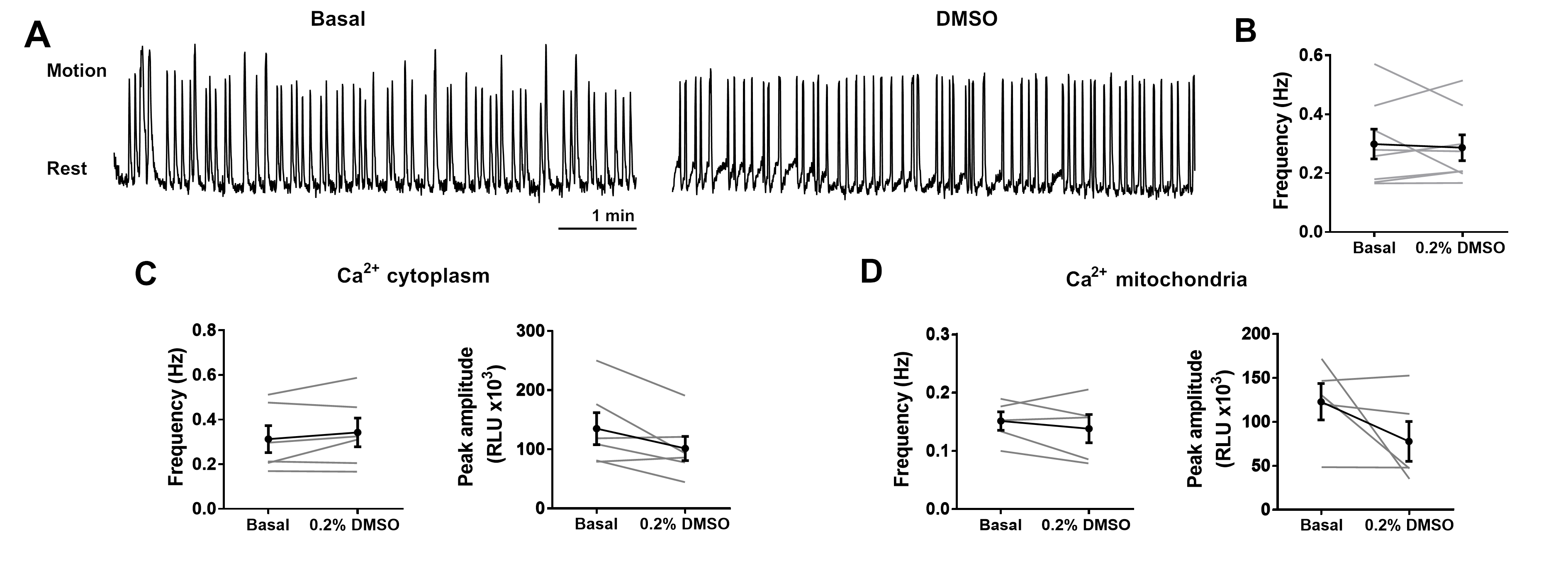

Supplement: Supplementary file 1 [file ijms-20-05409-s001.zip › Figure Supp2.tif]

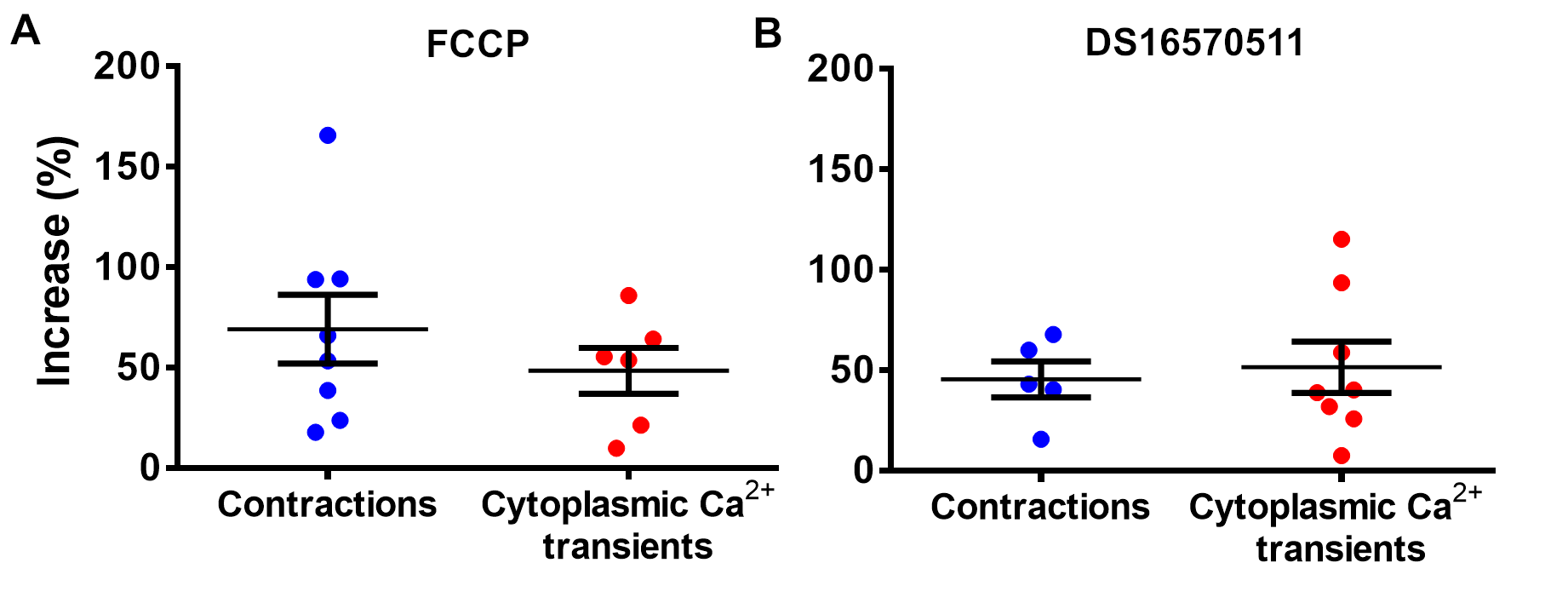

Supplement: Supplementary file 1 [file ijms-20-05409-s001.zip › Figure Supp3.tif]

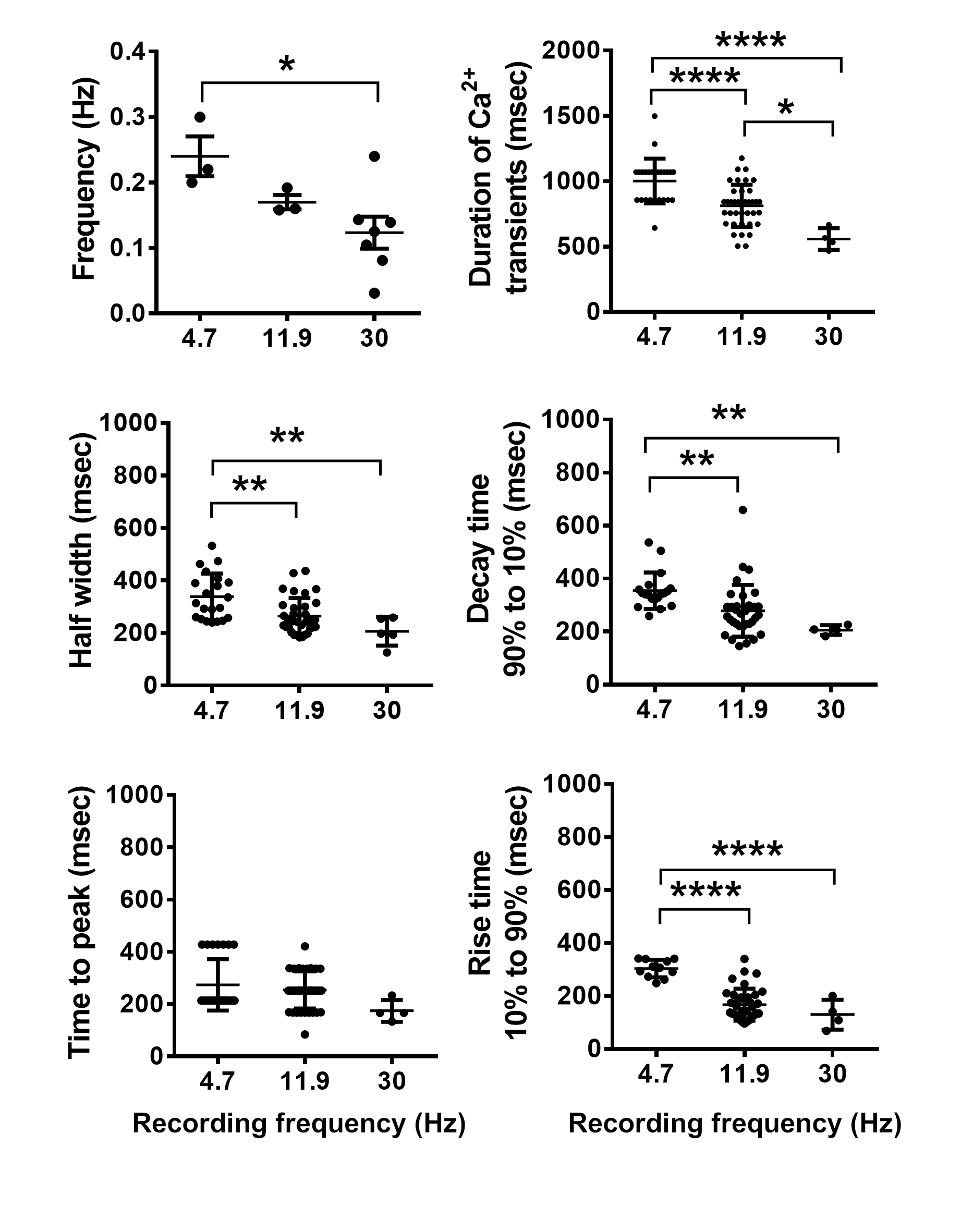

Supplement: Supplementary file 1 [file ijms-20-05409-s001.zip › Figure Supp4.tif]
